# Drone- and Paper-Based Analytical Devices: A Powerful Combination for the Colorimetric Detection of Tropospheric Ozone

**DOI:** 10.1021/acs.analchem.5c01889

**Published:** 2025-07-16

**Authors:** Pedro P. E. Campos, Habdias A. Silva-Neto, Lucas C. Duarte, João Flávio da Silveira Petruci, Wendell K. T. Coltro

**Affiliations:** † Instituto de Química, 67824 Universidade Federal de Goia’s, Goiânia, Goiás 74690-900, Brazil; ‡ Departamento de Química, 28117 Universidade Federal de Santa Catarina, Florianópolis, Santa Catarina 88040-900, Brazil; § Institute of Chemistry, 28119 Federal University of Uberlandia, Uberlândia, Minas Gerais 38400-902, Brazil; ∥ Instituto Nacional de Ciência e Tecnologia de Bioanalítica, Campinas, São Paulo 13084-971, Brazil

## Abstract

Ozone is a harmful atmospheric pollutant whose elevated concentrations (i.e., higher than 0.16 mg m^–3^) and prolonged exposure cause severe damage to the human respiratory system and negatively affect flora and fauna. Vertical ozone monitoring remains challenging due to the limitations of traditional sensors, which are bulky, expensive, and slow to provide results at the site of interest. To address this problem, there is a critical need for portable technologies that allow for rapid and efficient in situ detection. This study presents, for the first time, the integration of paper-based analytical devices (PADs) with a commercial drone to combine them for remote sampling and ozone colorimetric detection. The PADs were manufactured using a stencil-printing technique on chromatographic paper, with circular vinyl stencil masks (Ø = 5 mm) applied to define the detection areas on the paper. The hydrophobic barrier was created by depositing varnish resin onto the stencil/paper surface, with the masks removed after drying, resulting in PADs ready for use. As proof of concept, the paper detection zone surfaces were impregnated with potassium indigotrisulfonate (ITS) and polyethylene glycol (PEG), aiming to sample and detect gaseous ozone. The colorimetric method was performed using a desktop scanner to capture images, which were analyzed by graphical software to evaluate the resulting color intensity that varied from blue to colorless. A commercial ozone generator was used to optimize the method parameters. Parameters such as reaction time, reagent volume, and PEG concentration were optimized, resulting in a linear response range for ozone between 0.9 and 7.6 mg, with an *R* ^2^ of 0.996, and a limit of detection of approximately 0.25 mg. A customized holder was fabricated by 3D printing to ensure the attachment of PADs on the aerial drone platform. The system successfully monitored tropospheric ozone levels, recording 6.8 ± 0.7 mg during the dry season and 0.9 ± 0.1 mg during wet periods, with an in-flight sampling time of just 120 s. This innovative system has great potential to advance environmental monitoring, offering a portable, low-cost solution for remote and real-time ozone detection.

## Graphical abstract

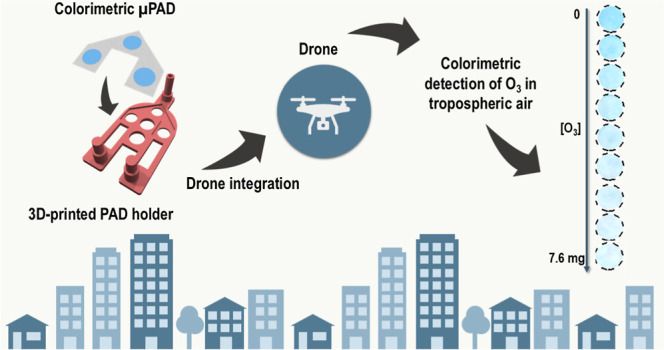

## Introduction

In a world where concerns about air quality and environmental impact are increasingly evident, monitoring atmospheric pollutants becomes vital. The troposphere, the layer of the atmosphere where we live, extends about 10–15 km above sea level and is composed of gases and particles.^1,2^ These can be directly emitted, considered primary, or formed as products of chemical reactions in the atmosphere, then regarded as secondary.^1,3^ Depending on the concentration levels, they become harmful to the entire ecosystem and are classified as pollutants. Ozone is a secondary tropospheric pollutant, found mainly in urban environments, originating from photochemical reactions involving nitrogen oxides and volatile organic compounds (VOCs).^4,5^ Prolonged exposure to high levels of tropospheric ozone seriously harms human health, especially the respiratory system, and harms ecosystems and various species of flora and fauna.^4,6–8^ Therefore, monitoring this gas is essential to identify and minimize its negative impacts.

Analytical chemistry still faces challenges in the in situ and remote quantification of tropospheric pollutants. Optical direct sensors provide more accurate detection in environmental samples by utilizing the strong absorption signature of ozone in the UV range.^9–14^ Methods based on chemiluminescence and fluorescence measurements have successfully demonstrated effectiveness in determining ozone in ambient air.^15–17^ However, these sensors are bulky, heavy, and expensive.^17–19^ Therefore, it is essential to develop portable and economically attractive analytical platforms to support effective air pollution reduction policies. Indirect methods are particularly promising because they use small, lightweight, and low-cost devices to collect the gaseous species.^20,21^ In this context, colorimetric detection has stood out in modern analytical chemistry, as it allows for rapid on-site detection^22^ through a chemical reaction between the target analyte and a specific colorimetric reagent.^23^ When the substance is present, the reaction results in a color change that can be observed with the naked eye or measured quantitatively using a spectrophotometer or by evaluating the image captured by scanners or smartphone cameras.^23–25^ Garcia et al.^26^ adapted a colorimetric detection method for ozone measurements. This approach was based on the reaction between ozone and the dye indigotrisulfonate (ITS). Ozone binds to indigo through its carbon–carbon double bond, generating an almost colorless product. The samples were analyzed by spectrophotometry, achieving a detection limit of 3.8 part-per-billion, considering an 8 h sampling period. Cerrato-Alvarez et al.^27^ created a passive sampler for the colorimetric determination of ozone, utilizing the reaction between ozone and ITS. The sampler consisted of a Teflon chamber containing a cellulose filter precoated with ITS, stored in a meteorological shelter. The authors employed a smartphone to capture the images, and the ozone determination was performed after processing and analyzing the digital images, achieving a limit of detection (LOD) of 3.3 μg/m^3^ during a 24 h exposure period.

Indirect colorimetric methodologies associated with paper-based analytical devices (PADs) can significantly expand their ozone monitoring potential when coupled with drones. This integration can enable efficient mapping of long-term average concentration variations over large geographic areas. In recent years, it has been increasingly adopted across various scientific and industrial sectors, providing creative and innovative solutions to complex challenges. According to the annual report by Drone Industry Insights, the global drone market is expected to reach $54.6 billion by 2030, with a constant compound annual growth rate of 7.7%.^28^ This growth reflects the versatility of these platforms in multiple fields, including military missions, rescue operations, precision agriculture, advanced logistics services, and indoor warehousing.^29–32^

Despite these advances, the use of drones in analytical chemistry is still in its early stages.^33^ Currently, their use is primarily focused on transporting sensors for remote monitoring of air quality^50^ and natural waters.^34,35^ Furthermore, there are no records to date of drones being used for ozone monitoring, especially with the use of PADs. In this study, we describe for the first time a suitable integration and assembly of PADs and a commercial drone for performing the sampling and colorimetric analysis of ozone. A three-dimensional (3D)-printed holder was manufactured with the aim of attaching multiple PADs to the drone platform. The proposed approach monitored ozone in the tropospheric air after the takeoff of the drone during a 120 s interval. We believe that this new approach can address the shortcomings of traditional ozone monitoring methods, providing more comprehensive coverage of ozone levels across geographic areas.

## Experimental Section

### Reagents and Instrumentation

Potassium ITS, PEG 400, potato starch, potassium iodide (KI), and sodium thiosulfate were obtained from Sigma-Aldrich (St. Louis, MO, USA). Sulfuric acid was purchased from Neon (Suzano, SP, Brazil). Analytical solutions were prepared using ultrapure water processed through a water purification system (Direct-Q 3, Millipore, Darmstadt, Germany) with resistivity equal to 18.2 MΩ cm without further purification. Adhesive vinyl, glass varnish, and Whatman chromatography paper were acquired from Imprimax (São Paulo, SP, Brazil), Acrilex (São Bernardo do Campo, SP, Brazil) and Cytiva (Washington, DC, USA), respectively. Polylactic acid filament (Ø = 1.75 mm) was obtained from 3DFila (Belo Horizonte, MG, Brazil).

Crafter printer, Prusa i3MK2 printer, and ozone generator (model GL-3189A) were purchased from Silhouette (Belo Horizonte, MG, Brazil), Prusa Research (Prague, Czech Republic), and Shenzhen Guanglei electronics Co., Ltd. (Shenzhen, Guangdong, China), respectively. The drone model Syma X8 and scanner HP model Scanjet G4050 were obtained from Syma toys (Shantou, Guangdong, China) and Hewlett-Packard Company (Palo Alto, California, USA), respectively.

### Fabrication of the Paper-Based Device and 3D-Printed Holder

For manufacturing the PADs, the stencil-printing approach was employed as described elsewhere.^36^ The Cameo software was used to create the desired layout of the PADs, featuring three circular detection zones (ϕ = 5 mm), as shown in Figure 1. Afterward, a self-adhesive sheet was fixed on the paper substrate surface and utilized to create the stencil mold using a cutting printer. Next, using a spatula, pure glass varnish was incorporated to the surface of the stencil mask/porous paper. After 5 min, the stencil mask was removed, and the PAD was ready to use, as illustrated in Figures 1A and S1 (available in the electronic Supporting Information, ESI).

**Figure 1:**
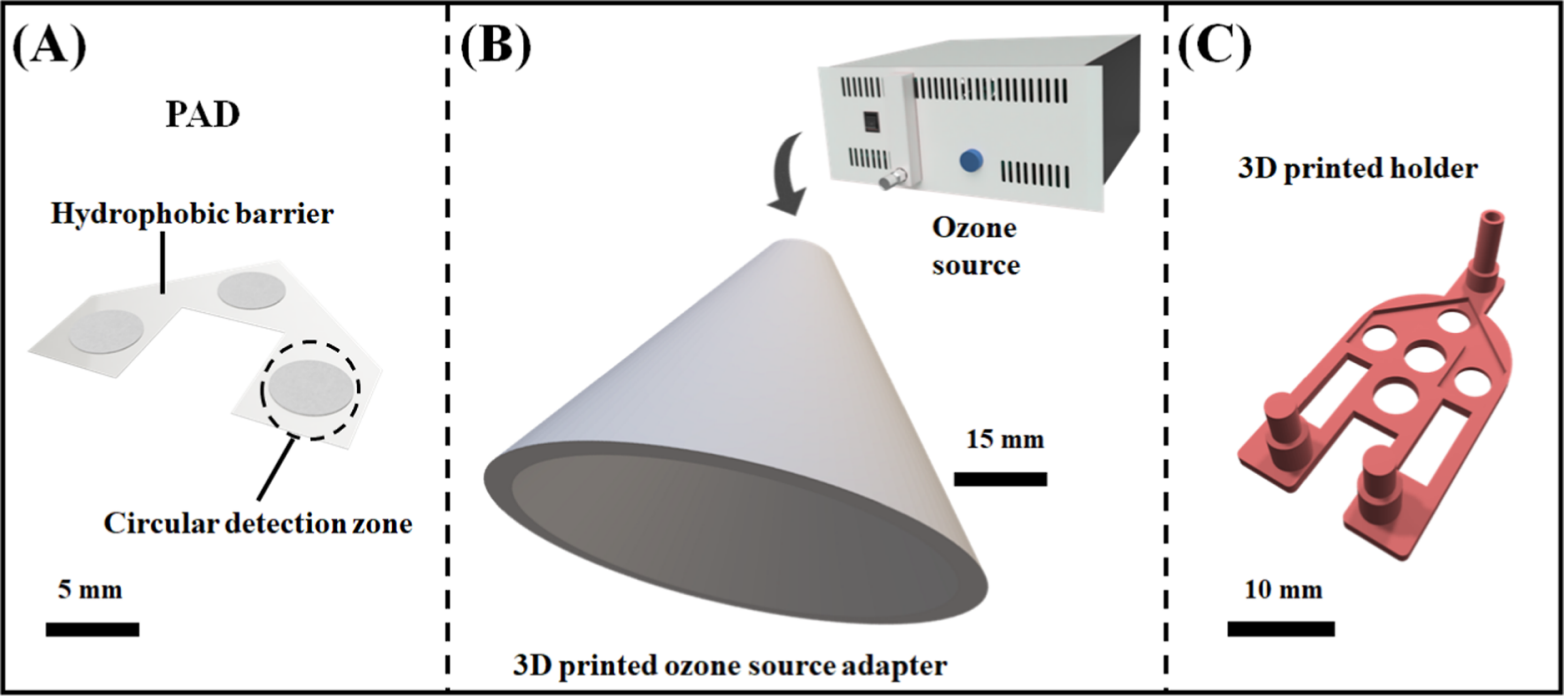
(A) Schematic illustration of PADs contending three different circular detection zones. Obtained projects that have been utilized for printing two different holders, (B) a device that can be used for inserting the PAD, and (C) a device to attach the PAD on drone.

Two distinct 3D-printed holders were also manufactured employing the additive manufacturing technique.^37,38^ (i) A plastic conical support with a base area of 15 cm and a height of 63 cm, was designed to couple PADs and attach in the ozone generation, and (ii) another plastic segment was created for assembling the PADs in the drone. The SolidWorks software was used to model the desired layouts and convert each project to an STL model. Additionally, the PrusaSlicer 2.3.0 software was employed for slicing the proposed project and setting the printing parameters, resulting in a G-code model. The selected printing conditions were height layer (0.2 mm), printing speed (40 mm s^–1^), printing style (cubic), infill (20%), bed temperature (60 °C), and nozzle temperature (210 °C). From Figure 1B, it is possible to see a schematic representation of the above-mentioned plastic holders.

### Attaching the Paper Device on 3D-Printed Holder

For performing benchtop colorimetric experiments, the PADs were attached inside the 3D-printed plastic cone (i), and the dispersion of ozone gas was started by using ozone generation. The PADs were integrated into a 3D-printed holder (ii) and then attached to the drone to take the air analysis utilizing the drone.

### Colorimetric Protocol and Optimization

The colorimetric measurement was based on ITS degradation in the presence of ozone gas, as described elsewhere.^27^ The reaction involves the sequential modification of paper zones with PEG 400, followed by the ITS reagent. The PEG concentration, reagent volume, reaction time, and color channel were carefully optimized to improve the sensing performance for ozone detection. For this purpose, the PEG concentration, the reagent volume, and the reaction time varied from 10 to 100% (v/v), 1–4 μL, and 1–8 min, respectively. Each paper zone was digitalized using an office scanner to obtain the colorimetric responses inside detection zones. The Corel Photo-Paint X8 software was used to obtain the pixel intensity from the region of interest (ROI) exploiting the RGB, CMYK, and cyan color channels.

### Analytical Performance

The desired paper zone was drop-casted with 1.5 μL of PEG 400 (50% v/v) for evaluating the analytical performance during benchtop experiments. After 5 min, a 1.5 μL aliquot of 1.0 mmol L^–1^ ITS was added into the circular zone. Next, the modified paper zone was attached in the 3D-printed holder and exposed to ozone gas at different times ranging from 15 to 120 s using a commercial ozone generation. Repeatability and reproducibility studies were also conducted employing an exposure time to ozone gas during 100 s. All experiments were performed in triplicate.

### Sampling and On-Site Colorimetric Detection of Ozone

Before the analysis, the modified PADs were attached in the 3D-printed holder and assembled on the drone. The drone was takeoff to an altitude ∼16 m and stayed there for ∼120 s, aspiring tropospheric gas at geographic coordinates (16°36′15.3″S 49°15′36.8″W). After landing, the PADs were removed and digitalized using an office scanner. Next, the cyan channel was utilized for obtaining the desired colorimetric signal (Figure 2).

**Figure 2:**
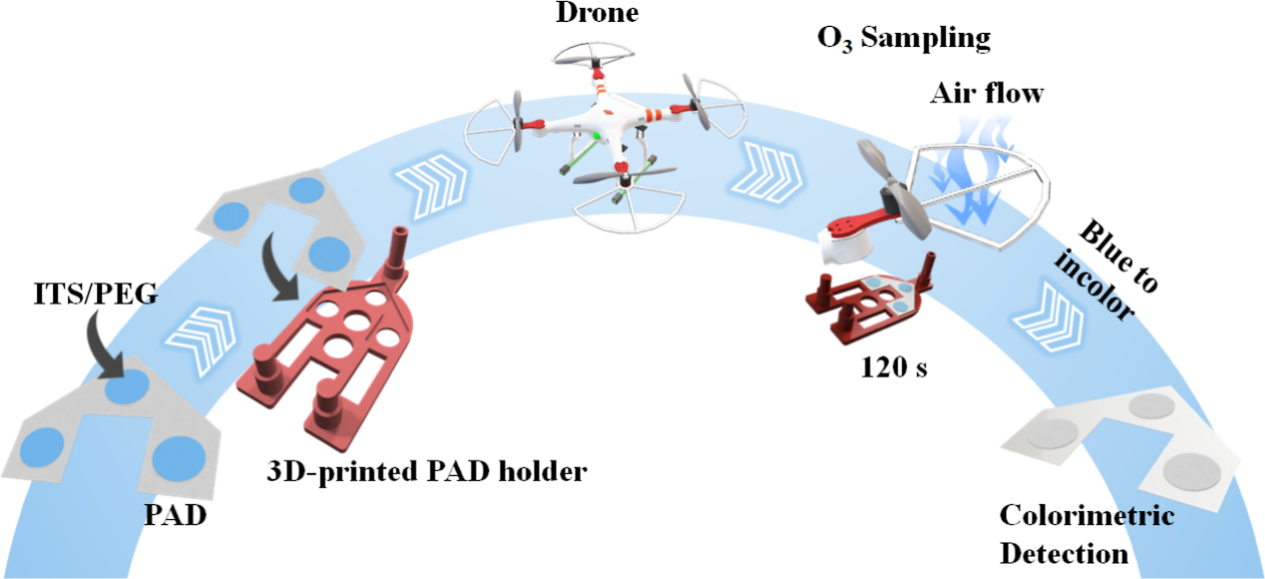
Schematic representation of the coupling process of the PAD-based sampling system to a drone for colorimetric ozone detection.

### Ozone Generator Characterization

To obtain the actual amount of ozone that is produced by the ozone generator, an iodometric titration procedure was used, as described elsewhere.^39,40^ For performing the colorimetric experiment, ozone gas was bubbled into a 25 mL solution of 2% (v/v) KI for approximately 1 h. Subsequently, a 4 mL aliquot of 12.4 mmol L^–1^ H_2_SO_4_ was added to the solution. Next, the desired solution was titrated with 1.21 mmol L^–1^ Na_2_S_2_O_3_, observing the complete color change to colorless by straw yellow. Then, another aliquot (2 mL of 1% (m/v) starch) was added to the KI solution, observing the color change from straw yellow to blue. The titration was finalized when the blue coloration became colorless. The actual mass of ozone gas produced by the generator was determined by calculating the amount of ozone that reacted with the KI in the solution.

## Results and Discussion

### Fabrication of the Analytical Device and Porous-Paper Modification

Paper-based devices have been utilized as portable analytical instrumentation due to advantages in terms of minimal consumption of sample and energy as well as their capacity to be used for on-site analysis.^41,42^ Additionally, the excellent performance of paper substrates for gas sampling has been successfully demonstrated, reinforcing their potential for this application.^43^ In this context, a PAD was developed using the stencil printing method,^36^ in which chromatographic paper and glass varnish were employed as hydrophilic and hydrophobic structures, respectively. To integrate this device with a drone, 3D printing was chosen due to its versatility and ability to produce objects with complex geometries quickly and cost-effectively, allowing for custom adaptations based on the design specifications.

The PAD, with a circular shape (ϕ = 5 mm), was designed to anchor the chromogen used for ozone detection and can be modified with two different reagents: PEG 400 and ITS, resulting in a colorimetric sensor for this gas. PEG was selected due to its ability to increase the hydrophilicity of the paper surface and promote a homogeneous distribution of reagents.^44^ These characteristics enhance sample interaction and improve reagent stability by reducing degradation and aggregation, thus resulting in more uniform and reproducible color development. Before starting the ozone detection by employing a combination of an analytical device and a drone, some experiments were carried out at the benchtop level. These experiments included the evaluation of PEG concentration, reagent volume, and color channel. The reaction time between the ITS and ozone gas was also studied, as carefully informed and discussed in the below section.

### Characterization and Optimization of Colorimetric Detection

For starting the colorimetric measurement, a commercial generator was utilized as a standard source of ozone gas. A well-known titration method was selected to determine the correct level of ozone produced by the generator,^45^ which revealed the value of 229 mg h^–1^. Regarding the colorimetric reaction, the presence of ozone gas can be confirmed through its reaction with ITS reagents previously impregnated on the paper surface.^27^ This chromogen originally presents a blue color, and when it reacts with ozone, the color changes to colorless (Figure 3A). To select the optimum PEG concentration to be added to PAD, colorimetric experiments were recorded employing varying the PEG concentration from 10 to 100% in the presence of ozone gas for 100 s (Figure 3).

**Figure 3:**
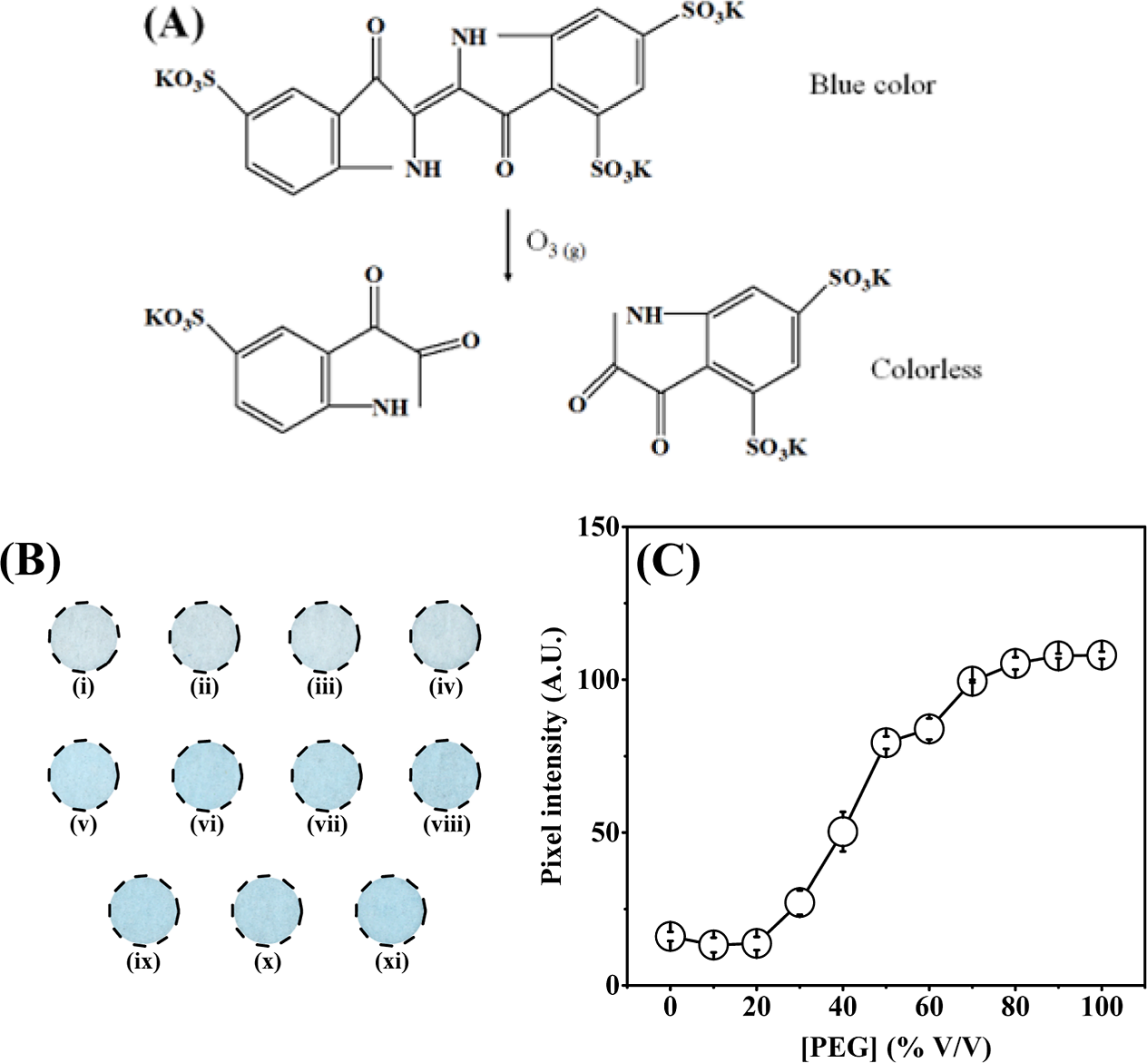
(A) A 2D representation of employed chemical reaction between ozone gas and ITS. (B) Circular detection zones obtained by using 11 different levels of PEG at % (v/v). (i) In the absence of PEG; (ii) 10%; (iii) 20%; (iv) 30%; (v) 40%; (vi) 50%; (vii) 60%; (viii) 70%; (ix) 80%; (x) 90%; and (xi) 100%. (C) Obtained histogram involving the pixel intensity response vs PEG concentration for ozone detection.

As demonstrated in Figure 3, the absence or low percentage of PEG compromises the colorimetric response for ozone. For PEG concentrations lower than 30%, the recorded pixel intensity was poor (pixel intensity ∼ 23 A.U.), while the PAD previously modified with higher concentrations of PEG exhibited enhanced performance, mainly when utilized 50% PEG (pixel intensity ∼ 79 A.U.). This behavior can be related to the suitable humidity that the PEG promotes on porous paper surfaces, increasing the color intensity signal for ozone.

Another aspect evaluated was the order of addition between PEG and ITS on the paper surface. From Figure S3, it is possible to see three different situations that have been explored, (i) the circular detection zone after inserting ITS before PEG, (ii) PEG before ITS, and (iii) *ex situ* mixture of PEG and ITS before inserting both on the paper surface. The best reaction occurs when PEG at 50% (v/v) concentration is drop-casted before ITS (increased the pixel response ∼ 1.9-fold). The probable reason to explain that behavior is that the presence of PEG before ITS onto the paper surface assured better uniformity and increased the probability of the desired reaction between ITS and ozone gas. If inserting ITS before PEG, the presence of PEG on the top of the paper surface can cover the ITS structure, reducing the performance of the desired reaction, which was observed through the low results in terms of pixel intensity and blue tonality.

Additionally, three other important sensing aspects were also studied: the color channel, volume of ITS solution, and reaction time. To optimize the color channel involving the desired reaction, three different filter colors (CMYK, cyan, and RGB) were utilized for constructing an analytical curve, as presented in Figure S2. After using the channels as filter color, the obtained responses in terms of slope and determination coefficient (*R* ^2^) were −0.53 and 0.91 for RGB and 0.41 and 0.99 for CMYK. When employed the cyan channel as a filter, the obtained slope and *R* ^2^ were 0.99 and 0.99, respectively, which presented an increase of ∼2.41-fold for sensitivity. It is also important to mention that the cyan channel presented suitable linearity when utilizing the color variation of ITS, avoiding the necessity to use external mathematical pretreatment.

For obtaining the ideal volume of ITS, aliquots (1–4 μL) were added to the reaction zone of PEG/PAD, and the achieved results are displayed in Figure 4. As it can be seen in Figures 4A,B, the addition of 3 μL of ITS onto PEG/PAD exhibited a better response regarding color uniformity and intensity (increasing 2.7-fold) when compared to a lower amount of ITS. The most reasonable hypothesis to justify this phenomenon is that the aliquot of 3 μL can cover all ROI to PAD, increasing the likelihood of chemical interaction between ITS and ozone.

**Figure 4:**
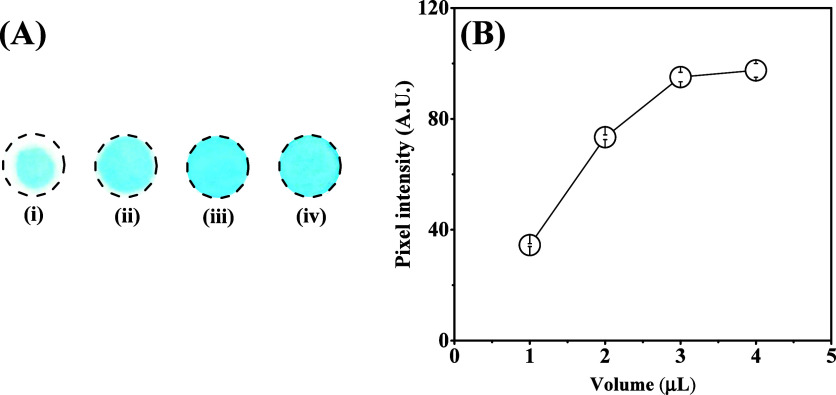
Optimization of the reagent volume. (A) Circular detection zones after using different volumes of reagent, (i) 1.0 μL, (ii) 2.0 μL, (iii) 3.0 μL, and (iv) 4.0 μL. (B) Resulting histogram indicating the effect of the ITS volume on the pixel intensity.

Different times ranging from 1 to 8 min were employed to select the adequate time necessary to dry the circular detection zones. As can be seen in Figure S4, it was observed that 4 min is the ideal time to perform before digitalizing the circular detection zone.

### Sensing Performance of Ozone

The analytical performance of the colorimetric assay for detecting ozone was successfully investigated, keeping all optimized conditions constant. As can be seen in Figure 5, the proposed approach revealed a suitable linear behavior (*R* ^2^ = 0.996) in the ozone mass range from 0.9 to 7.6 mg. The linear regression equation was pixel intensity (A.U) = 75.23–4.68 [O_3_: mg]. The LOD was calculated based on the ratio between 3.3 times the standard deviation values for the blank and the slope of the analytical curve, presenting a value equal to ∼0.25 mg.

**Figure 5:**
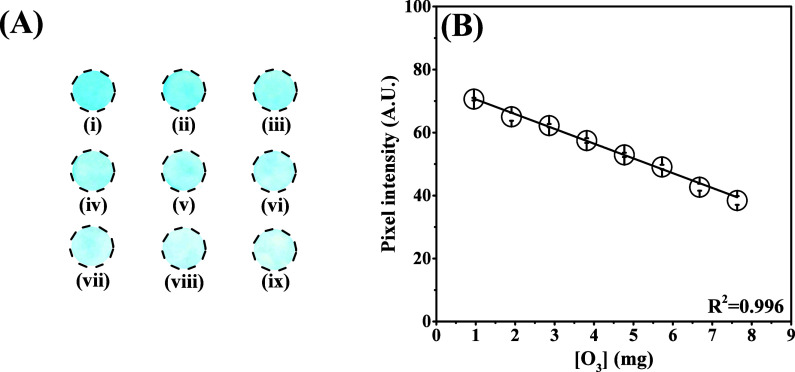
(A) Colorimetric detection zones obtained in the absence and presence of ozone. (B) Calibration curve obtained after exposing the PAD to increasing ozone levels: (i) blank, (ii) 0.9 mg, (iii) 1.9 mg, (iv) 2.9 mg, (v) 3.8 mg, (vi) 4.8 mg, (vii) 5.7 mg, (viii) 6.7 mg, and (ix) 7.6 mg. Sensing conditions: 50% (v/v) PEG and 1.0 mmol L^–1^ ITS.

The other two colorimetric experiments were recorded by employing three paper devices that were attached in a 3D-printed platform (repeatability assay) and three sensors attached in different 3D-printed platforms for the case of reproducibility. All experiments were recorded in the absence and presence of ozone gas for 100 s (Figure S5). The repeatability measurements presented suitable fluctuation in terms of pixel intensity, and the calculated value of relative standard deviation (RSD) was 3.4%. Similar behavior was observed for the reproducibility, which showed values of RSD ∼ 1.6%.

These experiments confirm that the proposed combination between PADs and 3D-printed holders can be utilized for measuring ozone gas. It is important to mention that the utilized process to obtain the colorimetric response does not employ sophisticated mathematical pretreatments. Recently, Cerrato-Alvarez et al.^27^ reported a combination between PAD and colorimetric reaction to measure ozone levels in tropospheric air. For sampling the gas, the authors employed a robust Ogawa passive sampler that was attached on a meteorological cabin. After aspiring air for 24 h, the PAD device was removed from the sampler. To obtain the analytical signal for ozone, the captured image of PAD was submitted to a mathematical algorithm, employing MATLAB software. The authors reported suitable sensing performance in terms of accuracy with RSD value of ca. 7%.

### Assembling the Paper-Based Device to Drone

To conduct on-site colorimetric analysis of ozone, three different PADs were attached in a 3D-printed holder, and that hybrid device was positioned on the drone, as schematically illustrated in Figure 2B. Before inserting that hybrid device on the drone, the circular zones were incorporated with the desired chromogen. The analytical tool was attached to the bottom of the drone’s engine by inserting a 3D-printed holder. This specific section of the drone was selected for attaching the devices because there is a high air flow during the flight time, assuring that the aspirated air had direct contact with the porous paper-device. This strategy enables air sampling without the need for a portable air pump. It is important to mention also that the drone’s engine can be promoted ∼25,000 rpm during the flight, which results in a suitable performance in terms of aspiring air.^46^

### Sampling Site and On-Site Analysis

Ozone gas can be generated at the troposphere by photochemical reactions when the precursors are available, including nitrogen oxides, VOCs, methane, and carbon monoxide. These precursors are originated from natural and anthropogenic ones.^47^ As proof of concept, the analytical approach herein proposed was used for sampling and identifying ozone gas at the troposphere. The desired samples were analyzed in a region with minimal trophogenic sources of ozone, which were collected in August 2023 (sample #1) and January 2024 (sample #2). For this purpose, the drone flew to a height of ∼16 m and aspired tropospheric air for 120 s.

Based on the data displayed in Figure 6, it is possible to infer that the obtained mass values for ozone gas were 6.8 ± 0.7 mg and 0.9 ± 0.1 mg for samples #1 and #2, respectively. When comparing the results obtained between all samples, it is possible to indicate also that a high amount of ozone gas was observed for sample #1. The decrease of ozone in sample #2 can be associated with increased levels of CO and NO_2_ gaseous.^18,48^ Siciliano et al.^49^ demonstrated that when air pollution is reduced, the ozone levels increase. The ozone levels increase as a consequence of the partial reduction of primary pollutants, including VOC and NO_*x*_. This hypothesis was confirmed during the lockdown promoted by COVID-19 in the city of Rio de Janeiro, Brazil.^49^

**Figure 6:**
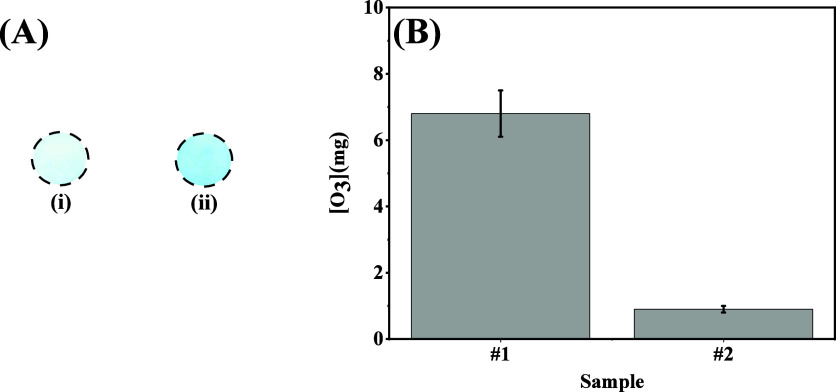
(A) Obtained ROI for circular detection zones after performing the colorimetric identification of ozone gas by using a drone, samples #1 (i) and #2 (ii). (B) Calculated amount of ozone at atmosphere air after utilizing the Cyan channel as filter color. All gas samples were collected after takeoff the drone at ∼16 m for 120 s.

## Conclusions

The development of a colorimetric sensor for gaseous sampling, embedded in drones using a 3D-printed holder, has proven to be a promising alternative tool for air quality monitoring, capable of detecting low levels of ozone in the atmosphere. The innovation of this device also lies in the combination of PEG with the chromogenic reagent ITS, which was essential for assuring adequate humidity in the reaction zones. Specifically, the concentration of PEG at 50% allowed for a significant increase in pixel intensity in the area of interest. Additionally, the choice of the cyan channel as a color filter proved to be more effective in terms of linearity and sensitivity, eliminating the need for additional mathematical treatments. The analytical performance of the device revealed a linear response range for ozone between 0.9 mg and 7.6 mg, with a coefficient of determination (*R* ^2^) of 0.996. The LOD calculated at ∼0.25 mg of ozone indicates suitable detectability for environmental monitoring applications, which was possible to determine the ozone concentration levels at the troposphere in rainy and dry seasons.

It is important to highlight that the city of Goiânia (Brazil) has no monitoring stations for atmospheric gases such as ozone; therefore, no official data on ambient ozone concentrations is available for comparison. Nevertheless, the primary objective of this study was to demonstrate the technical feasibility of integrating PADs with drones for environmental monitoring applications—which was successfully achieved based on the results obtained with the proposed methodology. In fact, as a screening tool, the proposed method is highly useful and can be employed by various agencies to estimate atmospheric pollutant concentrations in regions without monitoring infrastructure.

The integration of the system with a drone enabled air sample collection, eliminating the need for portable air pumps or other heavy and bulky equipment, facilitating monitoring in remote or hard-to-reach areas. These results highlight not only the high potential of the device for accurate ozone measurements but also its practical applicability in environmental monitoring scenarios, significantly contributing to research in atmospheric chemistry and pollution control. By using smartphones or digital light sensors to acquire color information from the PAD, we anticipate developing an analytical platform for in situ and online ozone monitoring based on the proposed approach. Furthermore, the sensor paves the way for interdisciplinary applications, such as the study of pollutant dispersion patterns and correlation with real-time meteorological data, enhancing the understanding of the impacts of ozone air quality and public health.

## Supplementary Material



## Notes

Additional images showing the resulting devices and complementary results associated with the optimization experiments (PDF)
