# Supplementary material for: Drone- and Paper-Based Analytical Devices: A Powerful Combination for the Colorimetric Detection of Tropospheric Ozone
Source: Anal Chem. 2025 Jul 16;97(29):15818–25. doi: 10.1021/acs.analchem.5c01889 (PMC12311897; doi:10.1021/acs.analchem.5c01889)
Supplement: Supplementary file 1 [file ac5c01889_si_001.pdf]

# Supporting Information

# Drone and paper-based analytical devices: A powerful combination for the colorimetric detection of tropospheric ozone

Pedro P.E Campos[a], Habdias A. Silva-Neto[a,b], Lucas C. Duarte[a], João Flávio da Silveira Petruci[c,]
and Wendell K. T. Coltro[a,d,*]

[a]Instituto de Química, Universidade Federal de Goiás, Goiânia, GO, 74690-900, Brazil

[b]Departamento de Química, Universidade Federal de Santa Catarina, Florianópolis, SC, 88040-900, Brazil

[c]Institute of Chemistry, Federal University of Uberlandia, Uberlândia, MG, 38400-902, Brazil

[d]Instituto Nacional de Ciência e Tecnologia de Bioanalítica, Campinas, SP, 13084-971, Brazil

## Table of Contents

**Figure S1.** Captured image of the proposed analytical device that was attached to the drone. **........S2**

**Figure S2.** Obtained results regarding the optimization study of color channels for ozone detection. (A) Experiments by using CMYK (A), cyan (B) and RGB (C) color systems.................................**S2**

**Figure S3.** Optimization of the sequence of deposition associated involving PEG and ITS. (A) Circular detection zones after inserting the reagent in that sequence (2º/1º) and after mixture ITP and PEG before adding to the PAD. (B) Histogram showing the relationship between color signal and the order of addition of PEG and ITS...........................................................................**S3**

**Figure S4.** Recorded experiments for finding the better time to digitalize the paper-based devices after performing the colorimetric reaction for ozone. (A) Circular detection after collecting the zones in different times (i) blank, (ii) 1 min, (iii) 2 min, (iv) 4 min, (v) 6 min and (vi) 8 min. (B) Obtained histogram involving the color signal and digitalizing time ..............................................**S3**

**Figure S5.** Recorded experiments for finding the better time to digitalize the paper-based devices after performing the colorimetric reaction for ozone. (A) Circular detection after collecting the zones in different times (i) blank, (ii) 1 min, (iii) 2 min, (iv) 4 min, (v) 6 min and (vi) 8 min. (B) Obtained histogram involving the color signal and digitalizing time .............................................**S4**

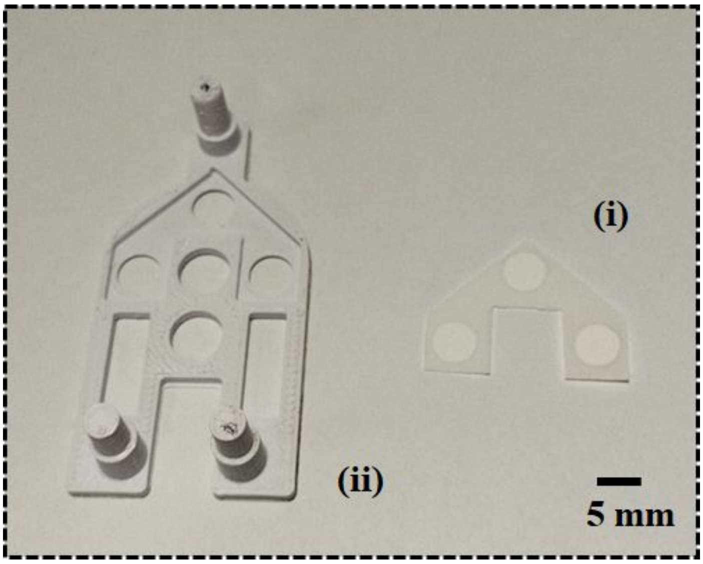


**Figure S1**. Captured image of the proposed analytical device that was attached to the drone.

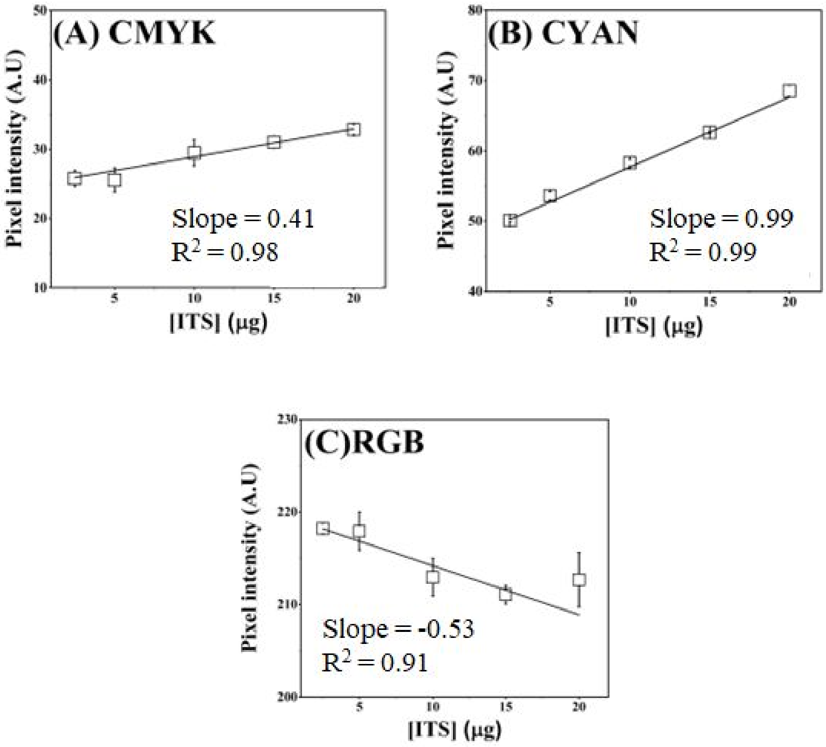


**Figure S2**. Obtained results regarding the optimization study of color channels for ozone detection. (A) Experiments by using CMYK (A), cyan (B) and RGB (C) color systems.

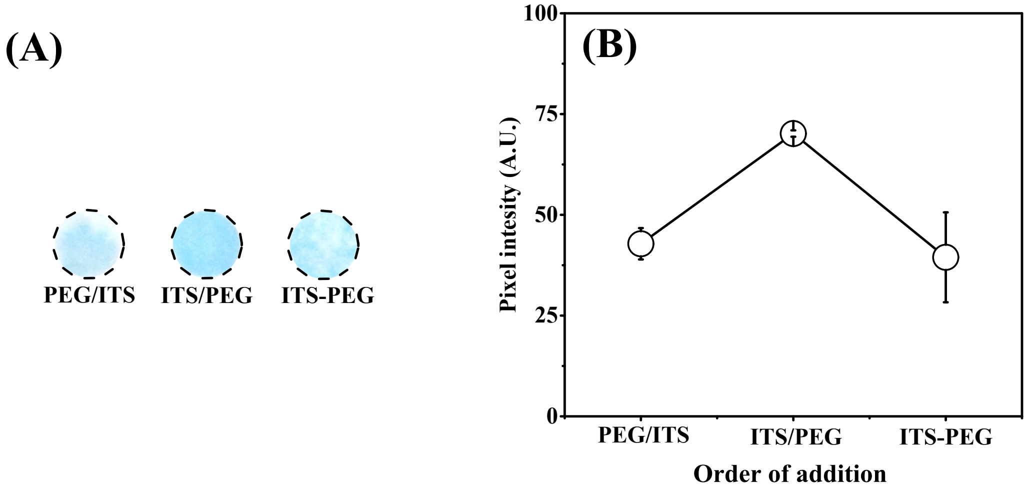


**Figure S3**. Optimization of the sequence of deposition associated involving PEG and ITS. (A) Circular detection zones after inserting the reagent in that sequence (2°/1°) and after mixture ITP and PEG before adding to the PAD. (B) Histogram showing the relationship between color signal and the order of addition of PEG and ITS.

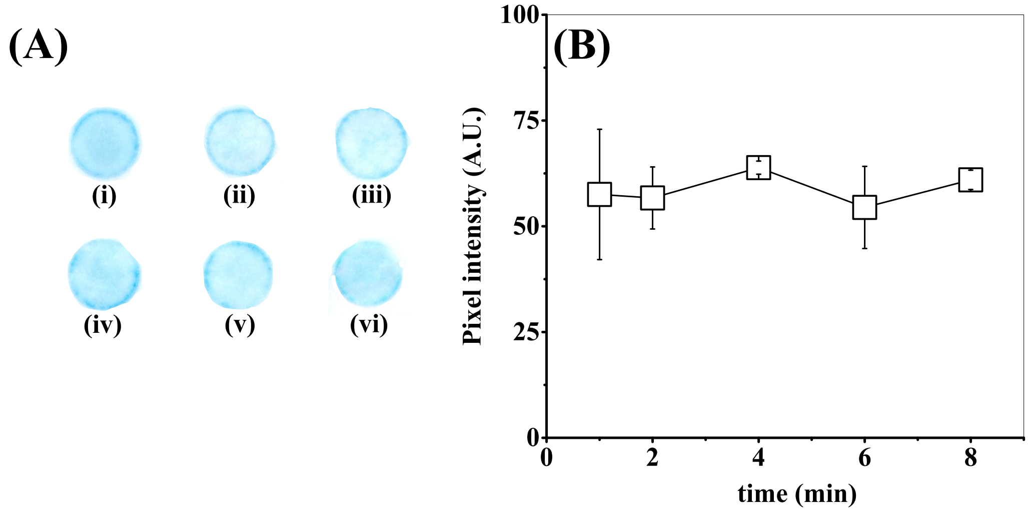


**Figure S4**. Recorded experiments for finding the better time to digitalize the paper-based devices after performing the colorimetric reaction for ozone. (A) Circular detection after collecting the zones in different times (i) blank, (ii) 1 min, (iii) 2 min, (iv) 4 min, (v) 6 min and (vi) 8 min. (B) Obtained histogram involving the color signal and digitalizing time.

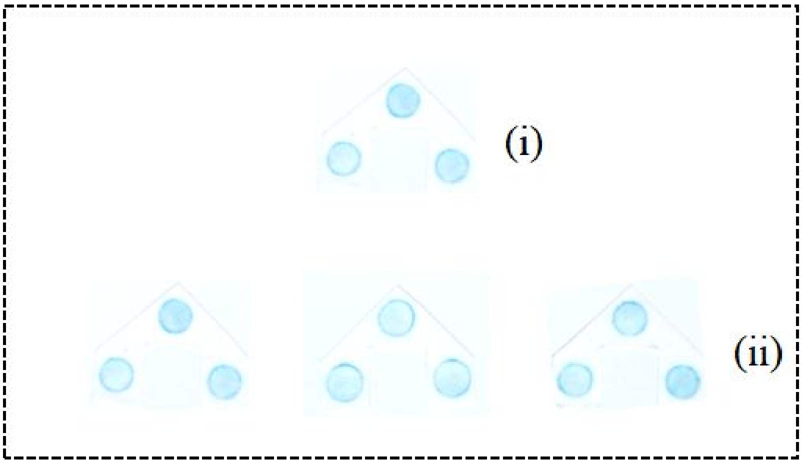


**Figure S5**. Obtained circular detection zones after performing the repeatability (i) and reproducibility studies (ii).

## AUTHOR INFORMATION

**Corresponding Author**

**Wendell K. T. Coltro:** Instituto de Química, Universidade Federal de Goiás, Goiânia, GO, 74690-900, Brazil and Instituto Nacional de Ciência e Tecnologia de Bioanalítica, Campinas, SP, 13084-971, Brazil. https://orcid.org/0000-0002-4009-2291; Email: wendell@ufg.br
